# Two-Photon Circularly Polarized Luminescence of Chiral Eu Complexes

**DOI:** 10.1021/jacs.3c05957

**Published:** 2023-11-08

**Authors:** Oliver G. Willis, Filippo Petri, Davide F. De Rosa, Alessandro Mandoli, Robert Pal, Francesco Zinna, Lorenzo Di Bari

**Affiliations:** † Department of Chemistry and Industrial Chemistry, University of Pisa, via Moruzzi, 13, 56124 Pisa, Italy; ‡ Department of Chemistry, Durham University, South Road, Durham DH1 3LE, U.K.

## Abstract

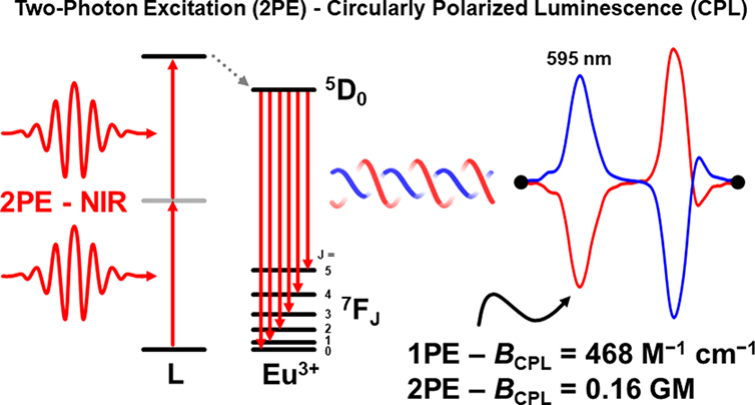

We report the synthesis of chiral lanthanide complexes with extended π conjugation for efficient circularly polarized luminescence (CPL) via two-photon excitation (2PE). The pyridine bis-oxazoline (PyBox) core provides the chiral Ln^3+^ environment, while the extension of the conjugated backbone through the pyridine 4-position with a phenylacetylene unit increases the two-photon absorption cross section. This work presents an important step toward the development of chiral systems displaying enhanced nonlinear optical properties, with potential applications in imaging and sensing, as well as in photodynamic therapy due to the selective excitation of molecules within a specific focal volume.

## Introduction

The recent resurgence of chiral systems that emit efficient circularly polarized luminescence (CPL) has attracted significant attention due to their potential applications in various fields, including photonics^1−4^ and optoelectronics.^5,6^ Trivalent luminescent lanthanide ions (Ln^3+^) are widely used as optical probes for imaging^7−9^ and luminescent sensing,^10−18^ as well as in security tags^19−25^ and OLED displays.^26−28^ However, the use of chiral emissive lanthanide complexes in biological imaging is limited by the need to excite them using higher-energy UV light, which is damaging to cells and has poor depth of penetration.^29^ One solution is to excite the sample using two identical half-energy photons (half the energy – double the wavelength), which enables deep tissue imaging with high spatial resolution, making it a valuable tool in fields such as neuroscience and biology.^30^ Some chiral emissive lanthanide complexes have been shown to undergo excitation by a two-photon excitation (2PE) process and to separately show CPL under regular one-photon excitation;^31,32^ however, only one recent report to date has combined both feats for molecular emitting species.^14^

One can quantify CPL emitters by their *B*_CPL_ value,^33^ which factors in the systems’ extinction coefficient (ε_λ_), quantum yield (ϕ), and dissymmetry value (*g*_lum_). For CPL spectra which contain multiple transitions within the same manifold, a more general definition of *B*_CPL_ must be applied (eq 1).^34^ The numerator evaluates the integral of an individual multiplet, where *a* and *b* denote the bounds of the transition, while the denominator considers the entire emission range.
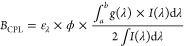
1

In the case of two-photon absorption (2PA), the probability that two photons will be absorbed simultaneously by a single molecule or material depends on the intensity of the light and the cross section of the material (σ^2^) with units of Goeppert–Mayer (GM) where 1 GM is 10^–50^ cm^4^ s photon^–1^.^35^ Thus, a modified *B*_CPL_ which accounts for the 2PE cross section can be used (eq 2) to assess the material’s brightness (2PE-*B*_CPL_).^14^
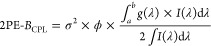
2

Given the low probability of the 2PE event, this value is expected to be much lower than the standard *B*_CPL_. Recently, the first example of CPL confocal microscopy was reported.^14^ It proved its ability in extracting information encoded in the circular polarization of the luminescent probe, which would be lost by using polarization-insensitive microscopy. For the reasons described above, 2PE-CPL probes would be significant assets for CPL confocal microscopy. However, the gap between technological abilities and the availability of suitable molecular systems for this task has yet to be filled.

**Scheme 1:**
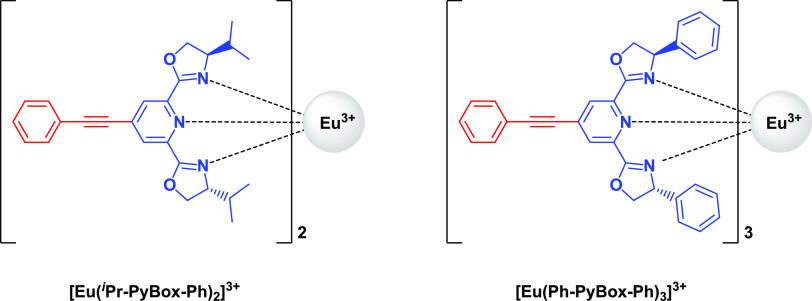
Two Sets of Eu Complexes Employed for 2PE-CPL

Here, we show that Eu complexes containing tailored chiral ligands can show efficient CPL via 2PE. A pyridine bis-oxazoline (PyBox) core was used to provide the chiral Ln^3+^ environment, with the conjugated backbone extended through the pyridine 4-position using a phenylacetylene unit. Following this strategy, enantiomer pairs of Eu^3+^ complexes for both the ^*i*^Pr and Ph-PyBox derivatives were synthesized (Scheme 1). On one hand, PyBox ligands have shown to be suitable chirality inducers in both homo- and heteroleptic complexes.^36−43^ On the other hand, Maury et al. showed that the strategy of ligands with extended conjugation and strong push–pull systems with varying charge-transfer character provides high 2PE cross sections in the case of dipicolinic acid derivatives.^31,32,44−54^

## Results and Discussion

PyBox derivatives can form lanthanide complexes with different stoichiometries depending on the substituents on position 4 of the oxazoline rings.^37^ The Eu^3+^ ion can accommodate up to three Ph-PyBox-Ph ligands but only two ^*i*^Pr-PyBox-Ph ligands due to an increased steric bulkiness. These results were confirmed by luminescent titrations (Figures S1 and S2) and elemental analysis and are all in accordance with the literature.^36,37^ Upon complexation of both ligands, a bathochromic shift of about 4000 cm^–1^ (40 nm) occurs for the lowest energy transition. This shift indicates a significant charge redistribution of the excited donor state which has been observed and calculated for similar systems and an increased charge-transfer character of the transition.^31,36^

The lifetimes of each complex were measured giving τ_obs_ values of 1.60 and 1.64 ms for [Eu(^*i*^Pr-PyBox-Ph)_2_]^3+^ and [Eu(Ph-PyBox-Ph)_3_]^3+^, respectively (Figure S3). The radiative component (τ_rad_) of the lifetime can be calculated using eq 3, thanks to the special nature of the ^5^D_0_ → ^7^F_1_ transition of Eu^III^ which possesses a purely magnetic dipole (MD) character.^55^
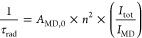
3Equation 3 contains *A*_MD,0_, the Einstein coefficient of the MD transition equal to 14.65 s^–1^,^55^ the refractive index (*n*), and a ratio of the total integrated emission *(I*_tot_) from the Eu (^5^D_0_) level to the ^7^F*_J_* manifold (*J* = 0–6) to the integrated intensity of the MD transition (*I*_MD_). The calculated τ_rad_ values for [Eu(^*i*^Pr-PyBox-Ph)_2_]^3+^ and [Eu(Ph-PyBox-Ph)_3_]^3+^ were 4.87 and 3.52 ms, respectively. The internal quantum efficiency (IQE, *Q*_Eu_^Eu^) can be calculated from the ratio of the observed lifetime (τ_obs_) and radiative lifetime (*Q*_Eu_^Eu^ = τ_obs_/τ_rad_) giving values of 33 and 47% for [Eu(^*i*^Pr-PyBox-Ph)_2_]^3+^ and [Eu(Ph-PyBox-Ph)_3_]^3+^, respectively. The external quantum efficiency (EQE, *Q*_Eu_^L^), measured using coumarin 153 (ϕ_r_ = 54.4% in ethanol)^56^ as the reference, gave values of 31 and 43% for the two complexes (eq S2), which correspond to sensitization efficiencies of 94 and 91% (η = *Q*_Eu_^L^/*Q*_Eu_^Eu^). A summary of these photophysical, as well as the chiroptical, results is collated in Table 1. The better EQE and IQE of the Ph-PyBox-Ph complex, compared to the ^*i*^Pr-PyBox-Ph, can be attributed to the more efficient protection of the Eu^3+^ core from possible quenchers such as solvent molecules, thanks to the increased steric bulk of having three coordinating Ph-PyBox-Ph ligands compared to two ^*i*^Pr-PyBox-Ph. Similar sensitization efficiencies between the ^*i*^Pr-PyBox-Ph and Ph-PyBox-Ph ligands are also congruent with predictions, as both complexes possess the same dominant chromophore and subsequent donor state. As known for closely related compounds,^36,57^ the high sensitization efficiency, close to 100%, is a direct result of the charge-transfer (CT) donor state of the conjugated ligands due to its push–pull nature. As calculated for a closely related arylacetylene-^*i*^Pr-PyBox lanthanide complex,^36^ the efficient ligand-to-Eu energy transfer is due to the low-energy and highly polarizable CT transition, with the transition dipole oriented parallel to the conjugated chromophore long axis. This has implications for the efficient 2PE described below.

**Table 1:** Summary of the Photophysical and Chiroptical Measurements of the Two Complexes Studied, Including the 2PE Cross Sections and both the 1PE and 2PE-*B*_CPL_ Results

complex	*ε*, M^–1^ cm^–1^ (λ, nm)	*τ*_obs_, ms	*τ*_rad_, ms	*Q*_Eu_^Eu^	*Q*_Eu_^L^	η	Δ*J*	|*g*_lum_| (λ, nm)	1PE-*B*_CPL_, M^–1^ cm^–1^	σ^2^, GM	2PE-*B*_CPL_, GM
[Eu(^*i*^Pr-PyBox-Ph)_2_]^3+^	49 968 (345)	1.60	4.87	0.33	0.31	0.94	1	0.08 (595)	468	17	0.16
							2	0.03 (615)	94		0.03
[Eu(Ph-PyBox-Ph)_3_]^3+^	74 827 (355)	1.64	3.52	0.47	0.43	0.91	1	0.04 (590)	141	22	0.04
								0.06 (593)	152		0.05
								0.09 (598)	570		0.17
							2	0.01 (613)	95		0.03
								0.01 (619)	33		0.01

The electronic circular dichroism (ECD) spectra of both ligands are characterized by weak structureless signals (Figure S4), which is a result of the inherently weak perturbation from the chiral centers distant in space from the chromophore transitions. The ^*i*^Pr-PyBox-Ph complex, which contains two ligands, shows a significantly different ECD spectrum with respect to the ligand but with a very little enhancement (Figure S5). This is a result of a weak coupling of the low-energy (pyridine–ethynyl–phenyl) transition.^36^ On the other hand, the Ph-PyBox-Ph complex which contains a more “packed” coordination environment shows a strong enhancement of the ECD intensities by a factor of 20 with respect to the ligand (Figure S5).

**Figure 1:**
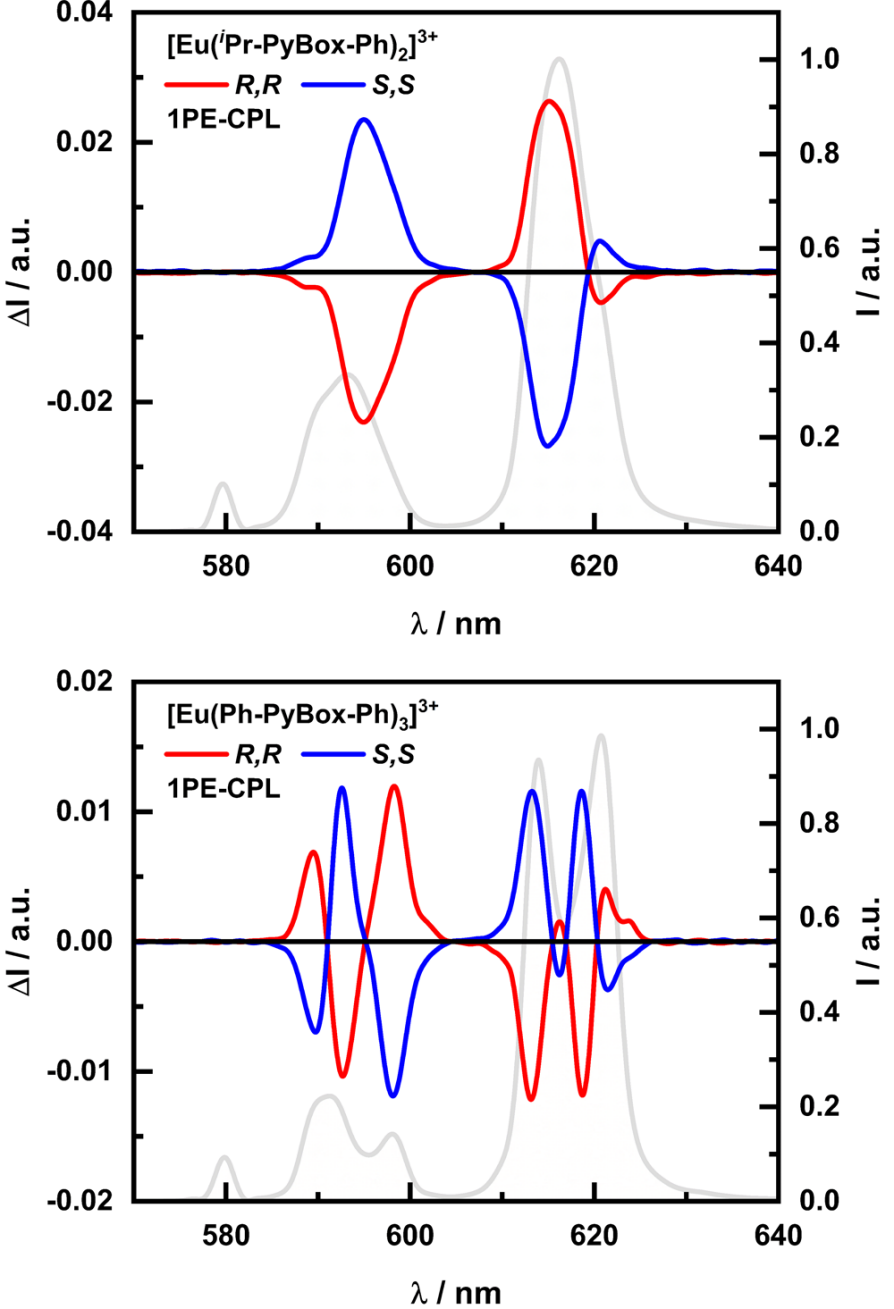
1PE-CPL spectra of [Eu(^*i*^Pr-PyBox-Ph)_2_]^3+^ (top) and [Eu(Ph-PyBox-Ph)_3_]^3+^ (bottom) with the normalized total emission traced in the background for both. Spectra were recorded in 0.01 mM acetonitrile solutions at room temperature under 340 nm excitation. Red solid line: *R,R* enantiomer and blue solid line: *S,S* enantiomer.

Upon excitation at 340 nm, both complexes exhibit unique CPL spectra that contain a varying number of oppositely signed bands (Figures 1 and S6). Mirror image spectra were recorded for each enantiomer. The [Eu(^*i*^Pr-PyBox-Ph)_2_]^3+^ complex shows two opposite major monosignate bands for the ^5^D_0_ → ^7^F_1_ (Δ*J* = 1) and ^5^D_0_ → ^7^F_2_ (Δ*J* = 2) transitions centered at 595 and 615 nm, respectively. These bands correspond to absolute dissymmetry values (|*g*_lum_|) of 0.08 (595 nm) and 0.03 (615 nm). The maximum |*g*_lum_| of 0.11 is obtained at 599 nm (Figure S7). On the other hand, the [Eu(Ph-PyBox-Ph)_3_]^3+^ complex displays a rich manifold with numerous bands of opposite signs. In fact, the Δ*J* = 1 manifold is fully resolved, with all three possible term-to-term transitions visible. The R,R enantiomer displays a +/–/+ signed pattern with peaks at 590, 593, and 598 nm, corresponding to *g*-values of +0.04, −0.06, and +0.09 (Figure S7). For both complexes, the dissymmetry values for the Δ*J* = 1 transition are comparable, despite [Eu(Ph-PyBox-Ph)_3_]^3+^ containing multiple overlapping bands of different signs, which can destructively convolute and weaken the emerging CPL emission (e.g., in the 610–630 nm region), especially if bandpass filters are used instead of monochromators. The Δ*J* = 2 for [Eu(Ph-PyBox-Ph)_3_]^3+^ is again rich in character, with potentially all five possible transitions being resolved. These bands occur at 613, 616, 619, 621, and 624 nm, with a −/+/–/+/+ pattern for the *R*,*R* complex and vice versa for the *S*,*S*, respectively. The two major CPL bands of the Δ*J* = 2 manifold at 613 and 619 nm both possess |*g*_lum_| values of 0.01 (Figure S7). The difference in the CPL spectra of [Eu(^*i*^Pr-PyBox-Ph)_2_]^3+^ and [Eu(Ph-PyBox-Ph)_3_]^3+^ depends on the different stoichiometries in the two cases, an overall different crystal field, and a different arrangement of the PyBox ligands.

See Table 1 for a full breakdown of the chiroptical and photophysical properties.

The CPL spectra of the two Eu complexes are similar in terms of signature to the ones previously reported by Di Bari et al.^37^ for homoleptic, nonmodified Eu/PyBox systems (namely, [Eu(^*i*^Pr-PyBox)_2_]^3+^ and [Eu(Ph-PyBox)_3_]^3+^). For both [Eu(^*i*^Pr-PyBox-Ph)_2_]^3+^ and [Eu(Ph-PyBox-Ph)_3_]^3+^ reported here, the CPL signature observed for 595 and 615 nm transitions is the same as those reported for [Eu(^*i*^Pr-PyBox)_2_]^3+^ and [Eu(Ph-PyBox)_3_]^3+^, respectively.^37^

In fact, both complexes containing the (*R,R*)-^*i*^Pr-PyBox based ligands are characterized by an overall ∓ signature for the Δ*J* = 1 and 2 transitions. An opposite overall pattern (−/+) is found for (*R,R*)-Ph-PyBox based systems, after accounting for the different spectral resolution achieved in this and the previous report^37^ (see Figure S8). This consistency in spectral sign suggests a similar coordination environment in the two series of complexes, consistent with the fact that the substitution in position 4 of pyridine does not significantly perturb the coordination mode of the PyBox ligands.

**Figure 2:**
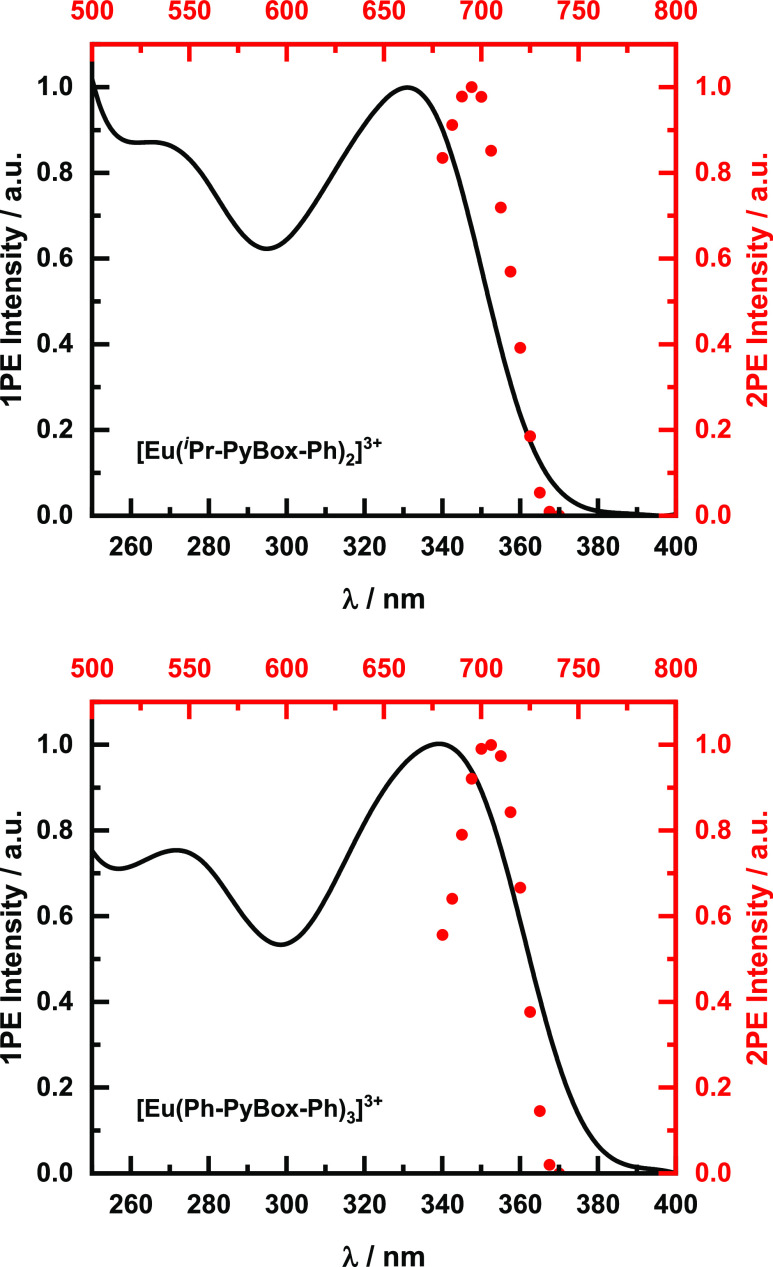
One-photon excitation (solid black line) and two-photon excitation (red dots) spectra (λ_em_ = 615 nm). Top: [Eu(^*i*^Pr-PyBox-Ph)_2_]^3+^. Bottom: [Eu(Ph-PyBox-Ph)_3_]^3+^.

Two-photon excitation (2PE) was achieved by irradiating the complexes with a tunable femtosecond pulsed laser (680–1300 nm, Coherent Discovery TPC, 100 fs, 80 MHz).^14^ Because of the nonlinear effect of 2PE (nondegenerate two-photon absorption), the 2PE maximum wavelength is usually not exactly double that of the 1PE absorption maximum (see Figure 2).^30,58^ The 2PE line shape is also inherently narrower than when excited with 1PE due to the quadratic relationship between the intensity of the 2PE excitation and the triggering of an emission event.^30,58^

**Figure 3:**
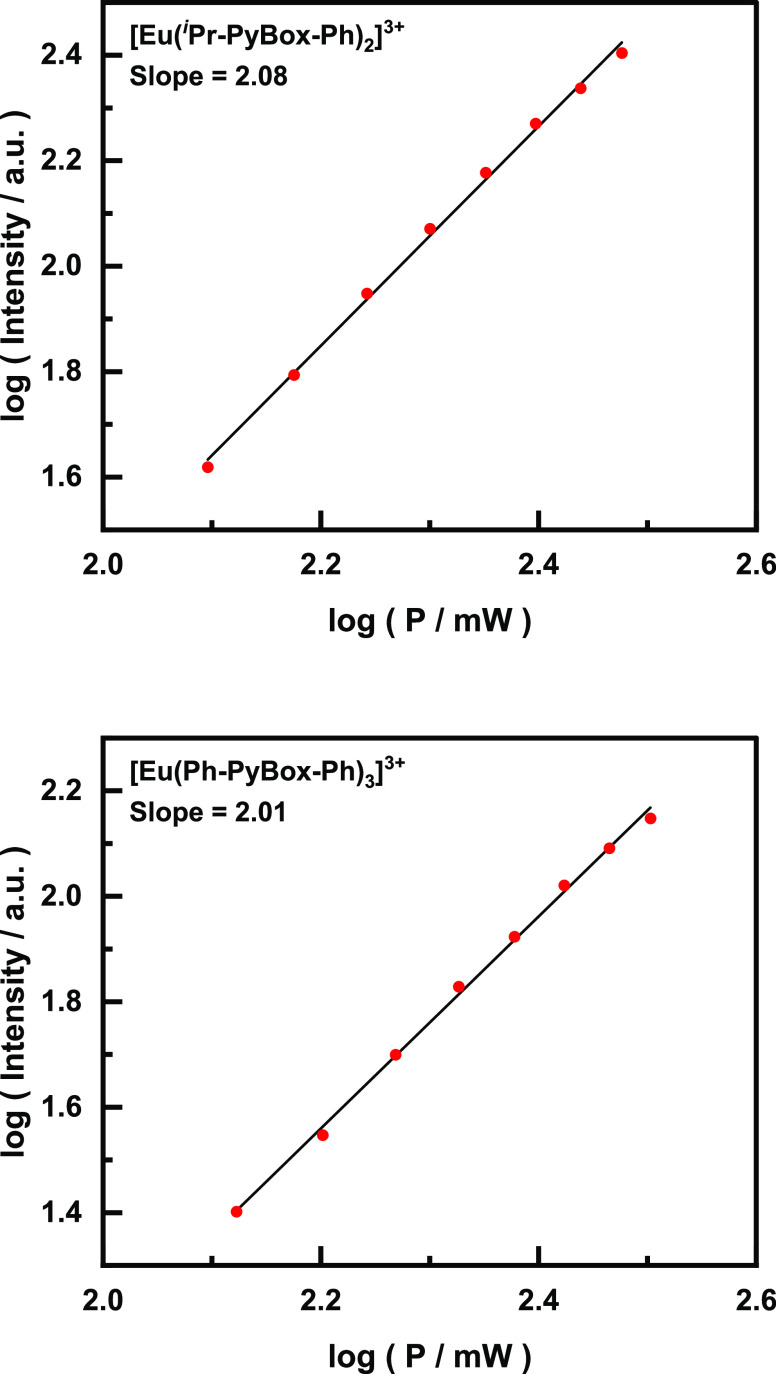
Log–log plot of the excitation-power dependence (red dots) of the 2PE-induced photoluminescence intensity. Top: [Eu(^*i*^Pr-PyBox-Ph)_2_]^3+^, slope 2.08. Bottom: [Eu(Ph-PyBox-Ph)_3_]^3+^, slope 2.01.

To confirm that excitation occurred via a 2PE event, the excitation-power dependence was recorded. As expected in the case of 2PE excitation, the intensity vs excitation-power log–log plots give a slope of two for both complexes (Figure 3).

**Figure 4:**
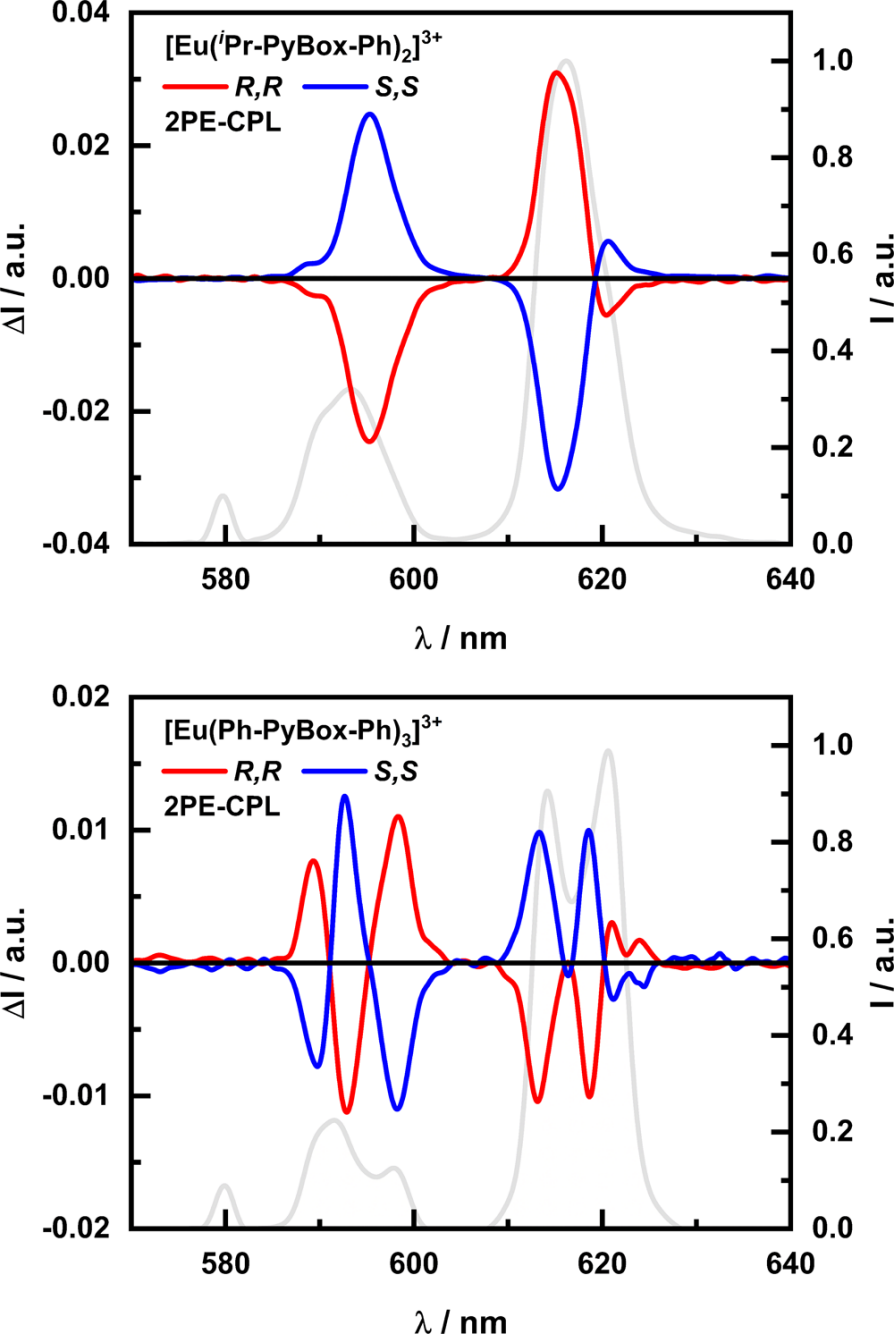
2PE-CPL spectra of [Eu(^*i*^Pr-PyBox-Ph)_2_]^3+^ (top, λ_ex_ = 700 nm) and [Eu(Ph-PyBox-Ph)_3_]^3+^ (bottom, λ_ex_ = 710 nm) with the normalized total emission traced in the background for both. Spectra were recorded in 0.01 mM acetonitrile solutions at room temperature. Red solid line: *R,R* enantiomer and blue solid line: *S,S* enantiomer.

Excitation of the two complexes using a pulsed laser at 700 nm for [Eu(Ph-PyBox-Ph)_3_]^3+^ and 710 nm for [Eu(^*i*^Pr-PyBox-Ph)_2_]^3+^ allowed for the measurement of 2PE-CPL using the same CPL spectrometer as the 1PE-CPL (Figure 4).^59^ The resulting 2PE-CPL spectra showed nearly identical manifolds and intensities to the 1PE-CPL spectra (Figure S9). The 2PE-CPL spectra only display the first three term-to-term transitions (Δ*J* = 0, 1, and 2) as a short pass filter was used at 650 nm to prevent the excitation light from reaching the detector.

The 2PE cross section was calculated using established procedures (eq S2) and with reference to rhodamine B (σ^2^ (700 nm) = 240 GM, σ^2^ (710 nm) = 180 GM)^60^ giving σ^2^ = 17 and 22 GM (1 GM = 10^–50^ cm^4^ s photon^–1^) for [Eu(^*i*^Pr-PyBox-Ph)_2_]^3+^ and [Eu(Ph-PyBox-Ph)_3_]^3+^, respectively.^35,58^ These values are in accordance with similar systems derived from dipicolinic acid.^31^ Overall, both complexes are intensely bright with 1PE-*B*_CPL_ values of up to 468 and 570 M^–1^ cm^–1^ from the ^5^D_0_ → ^7^F_1_ of both [Eu(^*i*^Pr-PyBox-Ph)_2_]^3+^ and [Eu(Ph-PyBox-Ph)_3_]^3+^, respectively. The calculated 2PE-*B*_CPL_ values for both the [Eu(^*i*^Pr-PyBox-Ph)_2_]^3+^ and [Eu(Ph-PyBox-Ph)_3_]^3+^ complexes gave values up to 0.16 and 0.17 GM from the most intense CPL transitions at 595 and 598 nm, respectively.

## Conclusions

In conclusion, the results discussed herein show how purposefully designed and tuned chiral ligands and the resulting Eu complexes can display CPL via 2PE. This goal is achieved via PyBox-type ligands with extended conjugation, which provide at the same time strong CPL, bright emission, and a sufficient 2PE cross section between 700 and 710 nm, summarized by a high 2PE-*B*_CPL_ value. This work provides one of the first examples of 2PE-CPL, opening the way to applications in polarized biological imaging applications and in the emerging field of CPL-microscopy. These compounds provide a modifiable scaffold, where substituents in the para-position with respect to the alkynyl phenyl ring may be added to tune the compatibility and affinity of the complexes with different media and cellular targets.

## Supporting Information Available

Compound preparation, instrumentation details, and additional spectra (PDF)
