# Supplementary material for: Two-Photon Circularly Polarized Luminescence of Chiral Eu Complexes
Source: J Am Chem Soc. 2023 Nov 8;145(46):25170–6. doi: 10.1021/jacs.3c05957 (PMC10683000; doi:10.1021/jacs.3c05957)
Supplement: Supplementary file 1 — ja3c05957_si_001.pdf [file ja3c05957_si_001.pdf]

Two-Photon Circularly Polarized Luminescence of Chiral Eu Complexes

Oliver G. Willis,[a]† Filippo Petri,[a] † Davide F. De Rosa,[b] Prof. Alessandro Mandoli,[a] Dr. Prof. Robert Pal,*[b] Dr. Francesco Zinna*[a] and Prof. Lorenzo Di Bari*[a]

[a] Department of Chemistry and Industrial Chemistry,
University of Pisa
via Moruzzi, 13, 56126, Pisa (Italy)
E-mail: francesco.zinna@unipi.it
E-mail: lorenzo.dibari@unipi.it

[b] Department of Chemistry,
Durham University,
South Road, DH1 3LE, Durham (UK)
E-mail: robert.pal@durham.ac.uk

† These authors contributed equally to this work.

We report the synthesis of chiral lanthanide complexes with extended π-conjugation for efficient circularly polarized luminescence (CPL) via two-photon excitation (2PE). The Pyridine bis-oxazoline (PyBox) core provides the chiral Ln^{3+} environment, while the extension of the conjugated backbone through the pyridine 4-position with a phenylacetylene unit increases the two-photon absorption cross-section. This work presents an important step towards the development of chiral systems displaying enhanced non-linear optical properties, with potential applications in imaging and sensing, as well as in photodynamic therapy due to the selective excitation of molecules within a specific focal volume.

Experimental Procedures

Reagents and general procedures

TLC analyses were carried out with Merck 60 F254 (0.2 mm) plates: a UV lamp (254 and 365 nm) was used for the detection of UV detectable organic compounds. The chromatographic purifications were performed with Macherey-Nagel flash grade silica gel (230-400 mesh) or using an Automated Chromatography system Isolera One 3.0™ with Biotage Sfär D-silica and Snap KP-Sil columns. The ^{1}H NMR and ^{13}C NMR spectra were recorded with a JEOL 400 MHz spectrometer. Chemical shifts are reported in ppm, with

frequency referencing made by setting the signal of the residual non deuterated solvent (^{1}H) or that of the deuterated solvent (^{13}C) to their recommended values.[1]

Unless otherwise specified, all the chemicals were used as obtained from the supplier (Sigma Aldrich, Tokio Chemical Industries, Carlo Erba Reagent). Triethylamine (TEA) was refluxed over CaH_2 for two hours, under an atmospheric pressure of dry N_2, and then distilled and stored in a dry Schlenk tube. MsCl was refluxed over P_4O_{10} at 80-90 mmHg for 2 hours and then distilled, at the same pressure. The MsCl was stored in a Schlenk tube under N_2 containing molecular sieves (MS) 4 Å. THF, DCM and Et_2O were obtained from a SPS 5/7 solvent purification system (Mbraun GmbH) and stored under an atmosphere of dry N_2. All the glassware used for the reactions under inert atmosphere were dried by heating under vacuum and then subjected to three vacuum-N_2 cycles. Unless otherwise specified, all the reactions were conducted under an atmosphere of dry N_2. Deuterated solvents were purchased from Deutero. Elemental analysis of CHNS were performed with a Vario MICRO cube CHNOS Analyzer (Elemental GmbH).

Synthesis of ligands

Dimethyl 4-bromo-2,6-pyridinedicarboxylate (1)

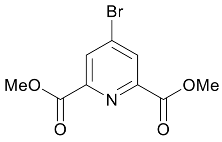

1

Following a literature procedure with modifications,[2,3] chelidamic acid (4.25 g, 25.43 mmol) and PBr_5 (25.00 g, 58.07 mmol) were heated for 24 hours at 80°C. The mixture was then cooled to 0°C and MeOH (50 mL) was added dropwise. The solution was concentrated under reduced pressure, then dissolved in $CHCl_3$ and washed with a saturated aqueous solution of NH_4Cl. The organic phase was dried (Na_2SO_4) and concentrated at reduced pressure to provide a white solid. The solid was recrystallized from hot heptane to give **1** (5.20 g, 75%). The NMR data were in agreement with those reported in the literature.[2,3] m.p. 159 °C; ^{1}H NMR (400 MHz,

$CDCl_3$): δ 8.45 (s, 2H), 4.02 (s, 6H); ^{13}C NMR (101 MHz, $CDCl_3$): δ 163.7, 149.7, 135.2 131.2, 53.8.

4-Bromo-*N*^{2},*N*^{6}-bis((*R*,*R*)-1-hydroxy-3-methylbutan-2-yl)pyridine-2,6-dicarboxamide (2-(*R*,*R*))

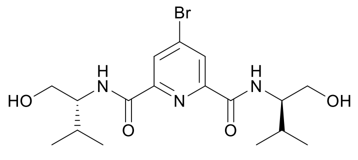

2-(*R*,*R*)

Following a literature procedure with modifications,[3,4] in a 50 mL Carius tube, **1** (1.32 g, 4.82 mmol) and (*R*,*R*)-valinol (0.99 mg, 9.63 mmol) were heated to 40 °C and stirred for 4 hours. The reaction was monitored by TLC (n-hexane/AcOEt). A white amorphous solid was obtained which was triturated with DCM and filtered to give **2-(*R*,*R*)** as a powdery white solid (1.43 g, 71% yield). The NMR data were in agreement with those reported in the literature.[3,4] R_f = −0.6 (n-hexane/AcOEt 3:7); m.p. 127 °C; $[\alpha]_D^{20}$ = −0.6 (c = 0.4 g/100 mL in $CHCl_3$); ^1H NMR (400 MHz, $CDCl_3$): δ 8.46 (s, 2H), 8.00 (d, J = 8.7 Hz, 2H), 3.95-3.85 (m, 6H), 2.73 (s, 2H), 2.11-2.01 (m, 2H), 1.03 (d, J = 6.8 Hz, 6H), 1.01 (d, J = 6.8 Hz, 6H); ^{13}C NMR (101 MHz, $CDCl_3$): δ 162.4, 151.2, 135.6, 127.7, 61.7, 57.7, 29.1, 19.8, 19.3.

4-Bromo-*N*^{2},*N*^{6}-bis((*S*,*S*)-1-hydroxy-3-methylbutan-2-yl)pyridine-2,6-dicarboxamide (2-(*S*,*S*))

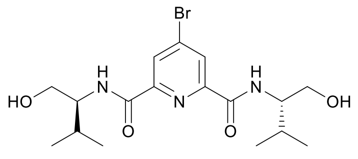

2-(*S*,*S*)

Obtained by the same method for **2-(*R*,*R*)**, by reacting **1** (1.28 g, 4.67 mmol) and (*S*,*S*)-valinol (0.96 g, 9.34 mmol) to give **2-(*S*,*S*)** as a white powder (1.53 g, 79%). The NMR data were in agreement with those reported in the literature.[3,4] R_f = 0.61 (n-hexane/AcOEt 3:7); m.p. 128 °C; $[\alpha]_D^{20}$ = +0.6 (c = 0.4 g/100 mL in $CHCl_3$); ^1H NMR (400 MHz, $CDCl_3$): δ 8.45 (s, 2H), 7.95 (d, J = 8.7 Hz, 2H), 4.00-3.80 (m, 6H), 2.66 (s, 2H), 2.10-2.04 (m, 2H), 1.02 (d, J = 6.8

Hz, 6H), 0.99 (d, J = 6.8 Hz, 6H); ^{13}C NMR (101 MHz, $CDCl_3$): δ 162.4, 151.1, 135.6, 127.7, 61.7, 57.7, 29.1, 19.8, 19.3.

4-bromo-*N*^{2},*N*^{6}-bis((*R*,*R*)-2-hydroxy-1-phenylethyl)pyridine-2,6-dicarboxamide (3-(*R*,*R*))

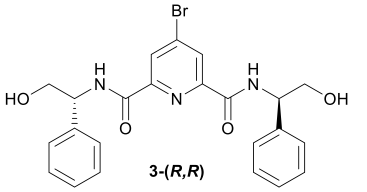

3-(*R*,*R*)

Following a literature procedure with modifications,[5] in a 50 mL Carius tube, **1** (1.31 g, 4.78 mmol) and (*R*)-(−)-2-Phenylglycinol (1.31 g, 9.56 mmol) were heated to 120 °C and stirred for 12 hours. After cooling, a colorless amorphous solid was obtained which, after purification by silica chromatography (n-hexane/AcOEt 4:6) provided **3-(*R*,*R*)** as a white crystalline solid (1.96 g, 85%). The NMR data were in agreement with those reported in the literature.[5] R_f = 0.36 (n-hexane/AcOEt 2:3); m.p. 135-137 °C; $[\alpha]_D^{20}$ = +0.7 (c = 0.4 g/100 mL in $CHCl_3$); ^1H NMR (400 MHz, $CDCl_3$): : δ 8.68 (d, J = 7.5 Hz, 2H), 8.39 (s, 2H), 7.41-7.26 (m, 10H), 5.22 (dd, J = 12.2, 4.9 Hz, 2H), 3.97 (d, J = 4.8 Hz, 4H), 3.04 (bs, 2H); ^{13}C NMR (101 MHz, $CDCl_3$): δ 163.2, 149.8, 138.7, 136.1, 129.1, 128.9, 128.0, 126.8, 65.8, 55.9.

4-bromo-*N*^{2},*N*^{6}-bis((*S*,*S*)-2-hydroxy-1-phenylethyl)pyridine-2,6-dicarboxamide (3-(*S*,*S*))

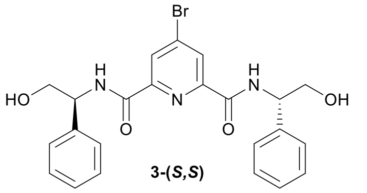

3-(*S*,*S*)

Obtained by the same method used for **3-(*R*,*R*)**, by reacting **1** (1.28 g, 4.67 mmol) and (*S*)-(+)-2-Phenylglycinol (1.28 g, 9.34 mmol) to give **3-(*S*,*S*)** as a white crystalline solid (1.43 g, 63%). The NMR data were in agreement with those reported in the literature.[5] R_f = 0.36 (n-hexane/AcOEt 2:3); m.p. 134-137 °C; $[\alpha]_D^{20}$ = −0.7 (c = 0.4 g/100 mL in $CHCl_3$); ^1H NMR (400 MHz, $CDCl_3$): δ 8.67 (d, J = 7.5 Hz, 2H), 8.37 (s, 2H), 7.41-7.25 (m, 10H), 5.22 (dd, J = 12.2,

4.9 Hz, 2H), 3.94 (d, J = 4.8 Hz, 4H), 3.03 (bs, 2H); ^{13}C NMR (101 MHz, $CDCl_3$): δ 163.2, 149.8, 138.7, 136.0, 129.0, 128.8, 128.0, 126.7, 65.8, 55.8.

(4*R*,4'*R*)-2,2'-(4-bromopyridine-2,6-diyl)bis(4-isopropyl-4,5-dihydrooxazole) (4-(*R*,*R*))

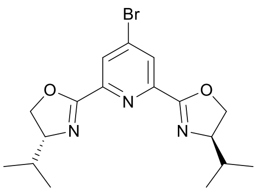

4-(*R*,*R*)

Following a literature procedure with modifications,[3,4] in a two-necked round bottom flask, **2-(*R*,*R*)** (0.98 g, 2.35 mmol), DMAP (28 mg, 0.23 mmol, 10% mol), MsCl (0.40 mL, 1.48 g/mL, 5.17 mmol), TEA (1.30 mL, 9.33 mmol) and DCM (12 mL) were stirred for 1 hour. The solution was then refluxed for 48 hours and monitored via TLC (n-hexane/AcOEt). The solution was washed with 10% NaOH and extracted with DCM (3 x 15 mL). The combined organic phases were dried (Na_2SO_4) and concentrated at reduced pressure. After column chromatography, **4-(*R*,*R*)** was recovered as a colorless crystalline solid (0.43 g, 48% yield). The NMR data were in agreement with those reported in the literature.[3,4] R_f = 0.34 (n-hexane/AcOEt 5:6); m.p. 132 °C; $[\alpha]_D^{20}$ = −0.4 (c = 0.4 g/100 mL in $CHCl_3$); ^{1}H NMR (400 MHz, $CDCl_3$): δ 8.35 (s, 2H), 4.53 (dd, J = 9.6, 8.7 Hz, 2H), 4.25 (m, 2H), 4.19 (m, 2H), 1.90 (m, 2H), 1.05 (d, J = 6.7 Hz, 6H), 0.94 (d, J = 6.7 Hz, 6H); ^{13}C NMR (101 MHz, $CDCl_3$): δ 161.6, 148.1, 133.8, 128.8, 73.7, 71.7, 34.4, 19.6, 18.9.

(4*S*,4'*S*)-2,2'-(4-bromopyridine-2,6-diyl)bis(4-isopropyl-4,5-dihydrooxazole) (4-(*S*,*S*))

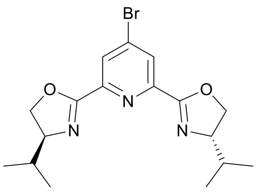

4-(*S*,*S*)

Obtained by the same method used for **4-(*R*,*R*)**, by reacting **2-(*S*,*S*)** (0.49 g, 1.18 mmol), DMAP (14 mg, 0.11 mmol, 10% mol), MsCl (0.20 mL, 2.58 mmol), TEA (0.70 mL, 5.02 mmol)

and DCM (8 mL) to give **4-(*S*,*S*)** (0.32 g, 71%). The NMR data were in agreement with those reported in the literature.[3,4] R_f = 0.34 (n-hexane/AcOEt 5:6); m.p. 135 °C; $[\alpha]_D^{20}$ = +0.4 (c = 0.4 g/100 mL in $CHCl_3$); ^{1}H NMR (400 MHz, CDCl3): δ 8.37 (s, 2H), 4.52 (dd, J = 9.6, 8.7 Hz, 2H), 4.25 (m, 2H), 4.10 (m, 2H), 1.85 (m, 2H), 1.03 (d, J = 6.7 Hz, 6H), 0.92 (d, J = 6.7 Hz, 6H); ^{13}C NMR (101 MHz, $CDCl_3$): δ 161.6, 148.1, 133.8, 128.8, 73.8, 71.6, 34.4, 19.6, 18.9.

(4*R*,4'*R*)-2,2'-(4-bromopyridine-2,6-diyl)bis(4-phenyl-4,5-dihydrooxazole) (5-(*R*,*R*))

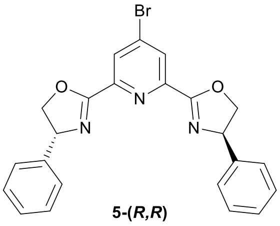

5-(*R*,*R*)

Following a literature procedure with modifications,[5] in a two-necked round bottom flask, **3-(*R*,*R*)** (0.50 g, 1.03 mmol), DMAP (13 mg, 0.11 mmol, 10% mol), TsCl (0.43 g, 2.27 mmol), TEA (0.57 mL, 4.09 mmol) and DCM (8 mL) were stirred for 1 hour. The solution was then refluxed for 48 hours and monitored via TLC (n-hexane/AcOEt). The solution was washed with 10% NaOH and extracted with DCM (3 x 15 mL). The combined organic phases were dried (Na_2SO_4) and concentrated at reduced pressure. After column chromatography, **5-(*R*,*R*)** was recovered as a colorless crystalline solid (0.31 g, 67% yield). The NMR data were in agreement with those reported in the literature.[5] R_f = 0.32 (n-hexane/AcOEt 6:4); m.p. 165-169 °C; $[\alpha]_D^{20}$ = +0.9 (c = 0.4 g/100 mL in $CHCl_3$); ^{1}H NMR (400 MHz, $CDCl_3$): δ 8.50 (s, 2H), 7.42-7.27 (m, 10H), 5.45 (dd, J = 10.3, 8.7 Hz, 2H), 4.93 (dt, J = 10.4, 7.1 Hz, 2H), 4.43 (dd, J = 11.7, 5.7 Hz, 2H); ^{13}C NMR (101 MHz, $CDCl_3$): δ 162.7, 149.8, 138.7, 138.6, 129.0, 128.5, 128.0, 126.8, 65.8, 55.9.

(4*S*,4'*S*)-2,2'-(4-bromopyridine-2,6-diyl)bis(4-phenyl-4,5-dihydrooxazole) (5-(*S*,*S*))

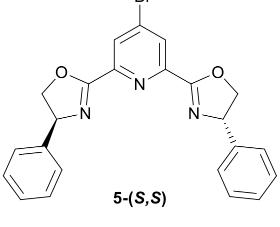

5-(*S*,*S*)

Obtained by the same method used for **5-(*R,R*)**, by reacting **3-(*S,S*)** (0.48 g, 0.99 mmol), DMAP (12 mg, 0.10 mmol, 10% mol), TsCl (0.42 g, 2.2 mmol), TEA (0.56 mL, 4.00 mmol) and DCM (8 mL) to give **5-(*S,S*)** as a colorless crystalline solid (0.28 g, 63%). The NMR data were in agreement with those reported in the literature.[5] R_f = 0.32 (n-hexane/AcOEt 6:4); m.p. 164-168 °C; $[\alpha]_D^{20} = -0.9$ (c = 0.4 g/100 mL in $CHCl_3$); ^1H NMR (400 MHz, $CDCl_3$): δ 8.49 (s, 2H), 7.43-7.27 (m, 10H), 5.43 (dd, J = 10.3, 8.7 Hz, 2H), 4.92 (dt, J = 10.4, 7.1 Hz, 2H), 4.43 (dd, J = 11.7, 5.7 Hz, 2H); ^{13}C NMR (101 MHz, $CDCl_3$): δ 162.7, 149.7, 138.7, 138.5, 128.9, 128.5, 128.0, 126.8, 65.8, 55.8.

(*R,R*)-^{i}Pr-PyBox-Ph

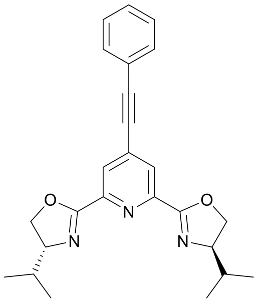

(*R,R*)-^{i}Pr-PyBox-Ph

Following a literature procedure with modifications,[6] a 50 mL two-necked round bottom flask, equipped with a magnetic stirrer and a condenser, was charged with **4-(*R,R*)** (104 mg, 0.27 mmol), phenylacetylene (90 µL, 0.93 g/mL, 0.82 mmol), CuI (0.5 mg, 2.7 µmol, 1% mol), $Pd(OAc)_2$ (3.1 mg, 13.7 µmol, 5% mol) and PPh_3 (7.2 mg, 27.4 µmol, 5% mol). After addition of TEA (0.3 mL, 2.5 mmol) and THF (5 mL), the solution was heated to 60°C and stirred for 4 hours. The reaction was monitored by TLC (n-hexane/AcOEt) and HPLC. Conc. NH_4Cl, was added to the solution, and the biphasic solution was vigorously stirred for an hour. The mixture was diluted with DCM, and the organic layer was separated and washed three times with a conc. NH_4Cl solution. After back-extraction of the aqueous phases with DCM, the combined organic extracts were dried (Na_2SO_4) and concentrated at reduced pressure. The resulting crude material was purified by column chromatography with an Isolera system. The product was purified further by triturating with Et_2O to give **(*R,R*)-^{i}Pr-PyBox-Ph** as a white solid (74 mg, 65%

yield). R_f = 0.29 (n-hexane/AcOEt 6:4); $[\alpha]_D^{20}$ = +0.5 (c = 0.4 g/100 mL in $CHCl_3$); ^1H NMR (400 MHz, $CDCl_3$): δ 8.27 (s, 2H), 7.58-7.48 (m, 2H), 7.40-7.35 (m, 3H), 4.53 (dd, J = 9.7, 8.3 Hz, 2H), 4.27-4.09 (m, 4H), 1.87 (h, J = 6.7 Hz, 2H), 1.05 (d, J = 6.7 Hz, 6H), 0.94 (d, J = 6.7 Hz, 6H); ^{13}C NMR (101 MHz, $CDCl_3$): δ 162.0, 147.2, 132.1, 129.6, 128.7, 127.4, 121.8, 95.8, 73.0, 71.2, 32.9, 19.1, 18.4; Elemental analysis calcd (%) for $C_{25}H_{27}N_3O_2$: C 74.79, H 6.78, N 10.47; found: C 74.40, H 6.68, N 10.28.

(*S*,*S*)-*^i*Pr-PyBox-Ph

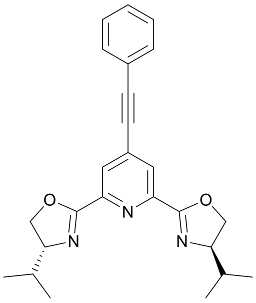

(*S*,*S*)-*^i*Pr-PyBox-Ph

Obtained by the same method used for **(*R*,*R*)-*^i*Pr-PyBox-Ph**, by reacting **4-(*S*,*S*)** (101 mg, 0.27 mmol), phenylacetylene (88 μL, 0.93 g/mL, 0.80 mmol), CuI (0.5 mg, 2.7 μmol, 1% mol), $Pd(OAc)_2$ (3.0 mg, 13.3 μmol, 5% mol) and PPh_3 (7.0 mg, 26.6 μmol, 5% mol), TEA (0.3 mL, 2.5 mmol) and THF (5 mL), to give **(*S*,*S*)-*^i*Pr-PyBox-Ph** as a white solid (36 mg, 34%). R_f = 0.29 (n-hexane/AcOEt 6:4); $[\alpha]_D^{20}$ = −0.5 (c = 0.4 g/100 mL in $CHCl_3$); ^1H NMR (400 MHz, $CDCl_3$): δ 8.29 (s, 2H), 7.57-7.51 (m, 2H), 7.43-7.34 (m, 3H), 4.54 (dd, J = 9.6, 8.3 Hz, 2H), 4.26-4.11 (m, 4H), 1.87 (m, 2H), 1.06 (d, J = 6.7 Hz, 6H), 0.95 (d, J = 6.8 Hz, 6H); ^{13}C NMR (101 MHz, $CDCl_3$): δ 162.1, 147.0, 132.7, 129.6, 128.7, 127.4, 121.8, 95.8, 73.4, 71.2, 32.7, 19.1, 18.5; Elemental analysis calcd (%) for $C_{25}H_{27}N_3O_2$: C 74.79, H 6.78, N 10.47; found: C 74.38, H 6.67, N 10.32.

(*R*,*R*)-Ph-PyBox-Ph

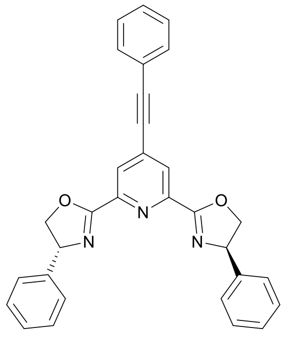

(*R*,*R*)-Ph-PyBox-Ph

Obtained by the same method used for **(*R*,*R*)-*i*Pr-PyBox-Ph**, by reacting **5-(*R*,*R*)** (100 mg, 0.22 mmol), phenylacetylene (73 μL, 0.93 g/mL, 0.67 mmol), CuI (0.4 mg, 2.2 μmol, 1% mol), $Pd(OAc)_2$ (2.5 mg, 11.1 μmol, 5% mol) and PPh_3 (5.8 mg, 22.3 μmol, 5% mol), TEA (0.3 mL, 2.5 mmol) and THF (5 mL), to give **(*R*,*R*)-Ph-PyBox-Ph** as a white solid (62 mg, 59%). R_f = 0.38 (n-hexane/AcOEt 6:4); $[\alpha]_D^{20}$ = −0.7 (c = 0.4 g/100 mL in $CHCl_3$); ^1H NMR (400 MHz, $CDCl_3$): δ 8.43 (s, 2H), 7.54-7.48 (m, 2H), 7.41-7.27 (m, 13H), 5.47 (dd, J = 10.3, 8.6 Hz, 2H), 4.94 (dd, J = 10.3, 8.6 Hz, 2H), 4.43 (t, J = 8.6 Hz, 2H); ^{13}C NMR (126 MHz, $CDCl_3$): δ 163.3, 147.1, 141.7, 133.6, 132.2, 129.7, 129.0, 128.7, 128.1, 128.0, 127.0, 121.7, 96.2, 85.7, 75.7, 70.5; Elemental analysis calcd (%) for $C_{31}H_{23}N_3O_2$: C 79.30, H 4.94, N 8.95; found: C 78.97, H 4.75, N 8.82.

(*S*,*S*)-Ph-PyBox-Ph

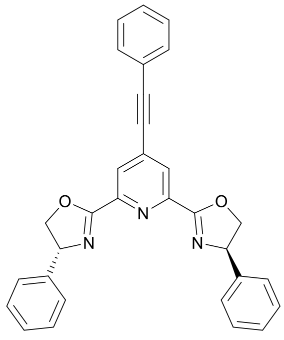

(*S*,*S*)-Ph-PyBox-Ph

Obtained by the same method used for **(*R*,*R*)-*i*Pr-PyBox-Ph**, by reacting **5-(*R*,*R*)** (108 mg, 0.24 mmol), phenylacetylene (80 μL, 0.93 g/mL, 0.72 mmol), CuI (0.5 mg, 2.4 μmol, 1% mol), $Pd(OAc)_2$ (2.7 mg, 12.1 μmol, 5% mol) and PPh_3 (6.3 mg, 24.1 μmol, 5% mol), TEA (0.3

mL, 2.5 mmol) and THF (5 mL), to give **(*S*,*S*)-Ph-PyBox-Ph** as a white solid (41 mg, 36%). R_f = 0.38 (n-hexane/AcOEt 6:4); $[\alpha]_D^{20}$ = +0.7 (c = 0.4 g/100 mL in $CHCl_3$); ^{1}H NMR (400 MHz, $CDCl_3$): δ 8.44 (s, 2H), 7.57-7.46 (m, 2H), 7.42-7.25 (m, 15H), 5.48 (dd, J = 10.3, 8.7 Hz, 2H), 4.95 (dd, J = 10.3, 8.7 Hz, 2H), 4.45 (t, J = 8.6 Hz, 2H); ^{13}C NMR (126 MHz, $CDCl_3$): δ 163.3, 147.0, 141.7, 133.6, 132.1, 129.7, 129.0, 128.7, 128.1, 128.0, 126.9, 121.7, 96.1, 85.7, 75.7, 70.4; Elemental analysis calcd (%) for $C_{31}H_{23}N_3O_2$: C 79.30, H 4.94, N 8.95; found: C 78.99, H 4.78, N 8.86.

Complexation

[Eu((*R*,*R*)-^{i}Pr-PyBox-Ph)$_2$] . 3[OTf]

In a two-necked 10 mL round-bottom flask equipped with a magnetic stirrer, **(*R*,*R*)-^{i}Pr-PyBox-Ph** (40 mg, 100 μmol) and $Eu(OTf)_3$ (30 mg, 50 μmol) in ACN (1.5 mL) were stirred for 24 hours. The reaction progress was followed by UV-Vis spectrophotometry. After complete complexation, the solution was concentrated at reduced pressure, leaving [Eu(**(*R*,*R*)-^{i}Pr-PyBox-Ph**)$_2$].3[OTF] (70 mg, 100%). $[\alpha]_D^{20}$ = +15.4 (c = 0.4 g/100 mL in $CHCl_3$); Elemental analysis calcd (%) for $C_{55}H_{60}EuF_9N_6O_{13}S_3$: C 46.12, H 4.22, N, 5.87, S, 6.72; found: C 46.62, H 4.11, N 5.98, S 6.81.

[Eu((*S*,*S*)-^{i}Pr-PyBox-Ph)$_2$] . 3[OTf]

In a two-necked 10 mL round-bottom flask equipped with a magnetic stirrer, **(*S*,*S*)-^{i}Pr-PyBox-Ph** (20 mg, 50 μmol) and $Eu(OTf)_3$ (15 mg, 25 μmol) in ACN (1.5 mL) were stirred for 24 hours. The reaction progress was followed by UV-Vis spectrophotometry. After complete complexation, the solution was concentrated at reduced pressure, leaving [Eu(**(*S*,*S*)-^{i}Pr-PyBox-Ph**)$_2$].3[OTF] (35 mg, 100%). $[\alpha]_D^{20}$ = −15.3 (c = 0.4 g/100 mL in $CHCl_3$); Elemental analysis calcd (%) for $C_{55}H_{60}EuF_9N_6O_{13}S_3$: C 46.12, H 4.22, N, 5.87, S, 6.72; found: C 45.89, H 4.23, N 6.14, S 6.54.

[Eu((*R*,*R*)-Ph-PyBox-Ph)$_3$] . 3[OTf]

In a two-necked 10 mL round-bottom flask equipped with a magnetic stirrer, **(*R*,*R*)-Ph-PyBox-Ph** (42 mg, 90 μmol) and Eu(OTf)$_3$ (18 mg, 30 μmol) in ACN (1.5 mL) were refluxed for 4 hours. The reaction progress was followed by UV-Vis spectrophotometry. After complete complexation, the solution was concentrated at reduced pressure, leaving [Eu(**(*R*,*R*)-Ph-PyBox-Ph**)$_3$].3[OTF] (52 mg, 100%). $[\alpha]_D^{20}$ = −140 (c = 0.4 g/100 mL in $CHCl_3$); Elemental analysis calcd (%) for $C_{99}H_{79}EuF_9N_9O_{15}S_3$: C 57.89, H 3.88, N 6.14, S 4.68; found: C 57.14, H 3.71, N 5.94, S 4.55.

[Eu((*S*,*S*)-Ph-PyBox-Ph)$_3$] . 3[OTf]

In a two-necked 10 mL round-bottom flask equipped with a magnetic stirrer, **(*S*,*S*)-Ph-PyBox-Ph** (23 mg, 60 μmol) and Eu(OTf)$_3$ (12 mg, 20 μmol) in ACN (1.5 mL) were refluxed for 4 hours. The reaction progress was followed by UV-Vis spectrophotometry. After complete complexation, the solution was concentrated at reduced pressure, leaving [Eu(**(*S*,*S*)-Ph-PyBox-Ph**)$_3$].3[OTF] (35 mg, 100%). $[\alpha]_D^{20}$ = +140 (c = 0.4 g/100 mL in $CHCl_3$); Elemental analysis calcd (%) for $C_{99}H_{79}EuF_9N_9O_{15}S_3$: C 57.89, H 3.88, N 6.14, S 4.68; found: C 57.25, H 3.69, N 6.01, S 4.48.

Instrumentation

UV-Vis/CD and Polarimetry measurements

UV-Vis spectra were recorded using a Jasco-V650 spectrophotometer in the spectral range of 200 to 450 nm. All samples were measured in 1 mM ACN solutions at room temperature with 0.01 cm cell length. The same solutions were used to record CD spectra using a J1500 spectropolarimeter in 0.01 cm optical glass cells and averaging 16 accumulations. The optical rotation was measured with a Jasco DIP360 digital polarimeter in a 10 cm polarimetric cell, using the Sodium lamp's D line.

Quantum yield measurements

Luminescence quantum yields (or External Quantum Efficiency, EQE, Q_x^L, ϕ_x) were measured with a Horiba Jobin–Yvon Fluorolog®-3 spectrofluorometer equipped with a 450 W Xenon arc lamp, Hamamtsu R928P detector, double-grating excitation, and single-grating emission monochromators. Fluorescence quantum yields were measured in ACN relative to coumarin 153 (ϕ_r = 54.4% in ethanol).[7]

$$\phi_x = \phi_r \cdot \frac{A_r(\lambda)}{F_r \cdot n_r^2} \cdot \frac{F_x \cdot n_x^2}{A_x(\lambda)} \qquad \textbf{(Eq S1)}$$

In Eq S1, A is the absorbance at the excitation wavelength, n is the refractive index and F the luminescence integrated intensity. The indices r and x stand for reference and sample respectively. The absorption and excitation wavelengths for both $[Eu(^{i}Pr\text{-}PyBox\text{-}Ph)_2]^{3+}$ and $[Eu(Ph\text{-}PyBox\text{-}Ph)_3]^{3+}$ were 360 nm.

Lifetime measurements

The observed lifetimes (τ_{obs}) were measured using a Perkin Elmer LS55 spectrometer using FL Winlab (3.1) software, with the following parameters: Gate time 0.1 ms, excitation slit width 10 nm, emission slit width 5 nm, minimum delay 0.1 ms, maximum delay 3 ms, delay step 0.1 ms and recorded in triplicates. The decay profiles were fitted using a mono-exponential function.

1PE-CPL

CPL was measured with a home-built (modular) spectrometer.[8] The excitation source was a broad band (200 – 1000 nm) laser-driven light source EQ 99 (Elliot Scientific). Sample PL emission was collected perpendicularly to the excitation direction. The emission was fed through a photoelastic modulator (PEM) (Hinds Series II/FS42AA) and through a linear sheet polarizer (Comar). The light was then focused into a second scanning monochromator (Acton SP2155) and subsequently on to a photomultiplier tube (PMT) (Hamamatsu H10723 series). The detection of the CPL signal was achieved using the field modulation lock-in technique. The electronic signal from the PMT was fed into a lock-in amplifier (LIA, Hinds Instruments

Signaloc Model 2100). The reference signal for the lock-in detection was provided by the PEM control unit. The monochromators, PEM control unit and LIA were interfaced to a desktop PC and controlled by a custom-written Labview2014 graphic user interface. The LIA provided two signals, an AC signal corresponding to (I_L- I_R) and a DC signal corresponding to (I_L + I_R) after background subtraction. The emission dissymmetry factor was therefore readily obtained from the experimental data, as 2 AC/DC. Spectral calibration of the scanning monochromator was performed using a Hg-Ar calibration lamp (Ocean Optics). A correction factor for the wavelength dependence of the detection system was constructed using a calibrated lamp). The measured raw data was subsequently corrected using this correction factor. The validation of the CPL detection systems was achieved using light emitting diodes (LEDs) at various emission wavelengths. The LED was mounted in the thermally stabilized sample holder (25 ºC, Thor labs CVH100) and the light from the LED was fed through a broad band polarizing filter and λ/4 plate (Ocean Optics) to generate circularly polarized light. Prior to all measurements, the λ/4 plate and a 650 nm LED were used to set the phase of the lock-in amplifier correctly. The emission spectra were recorded with 0.5 nm step size and the slits of the detection monochromator were set to a slit width corresponding to a spectral resolution of 0.25 nm. CPL spectra (as well as total emission spectra) were obtained through an averaging procedure of several scans.

2PE-CPL

Two photon excitation CPL spectroscopy was achieved by coupling (beam routing using mirrors, Thor Labs BB1- E03) a tunable femtosecond pulsed laser (680 – 1300 nm, Coherent Discovery TPC, 100 fs, 80 MHz) to the pre-existing CPL spectrometer detailed above.[9] Initial proof of concept two photon spectroscopy has been achieved by perpendicularly mounting an Ocean Optics HR2000Pro (2048-pixel linear CCD Sony ILX5 chip, 200 µm slit, H3 grating, 350 – 850 nm spectral region) spectrometer as a 'third arm' to the Discovery TPC laser. The laser beam was focused onto the center of the 1 cm path thermally stabilized sample holder (25 ºC, Thor labs CVH100) by a dedicated ultrafast laser lens (Edmund Optics 11711, 50 mm focal

length). The spectrometer has also been equipped with a perpendicularly mounted 365 nm LED (nichia, 500 mW) and been operated using a modified version of the above-mentioned custom time resolved detection and accumulation algorithm written in Labview2013 program. In order to eliminate unwanted artefacts associated with stray light from 2PE each spectrometer has been equipped with a rotating filter wheel (Thor Labs, CFW6) housing an LP420 (Comar Optics, for 365 nm UVLED excitation) and SP650 and SP700 (Edmund Optics, 8472 and 8474 for MP excitation) filters.

Cross section determination

The 2PE cross sections (σ^2) of the complexes were measured according to established procedures and using Eq S2.[10,11]

$$\sigma_x^2 = \sigma_r^2 \cdot \frac{\phi_r \cdot C_r \cdot n_r}{F_r} \cdot \frac{F_x}{\phi_x \cdot C_x \cdot n_x} \qquad \textbf{(Eq S2)}$$

Where x is the sample, *r* is the reference, ϕ is the total emission quantum yield of the compound, C is the concentration, *n* the refractive index and F is the integrated PL spectrum. The cross sections were calculated with reference to Rhodamine B in ethanol (σ^2 (700 nm) = 240 GM, σ^2 (710 nm) = 180 GM).[12]

Results and Discussion

Additional spectra

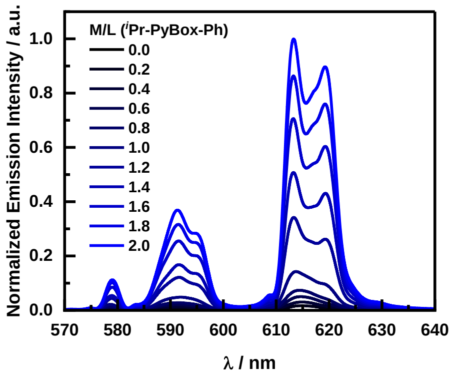

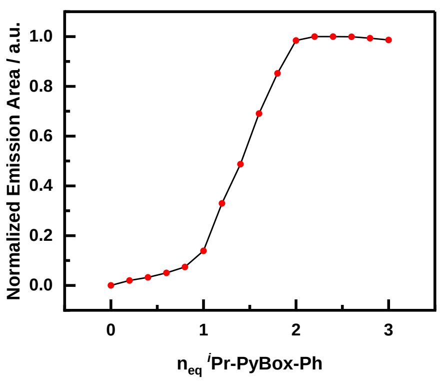

Figure S1. Left: The normalized emission intensity of $Eu(OTf)_3$ with various equivalents of ^{i}Pr-PyBox-Ph. Right: The isotherm titration curve from the normalized emission integrated area at varying equivalents of ^{i}Pr-PyBox-Ph.

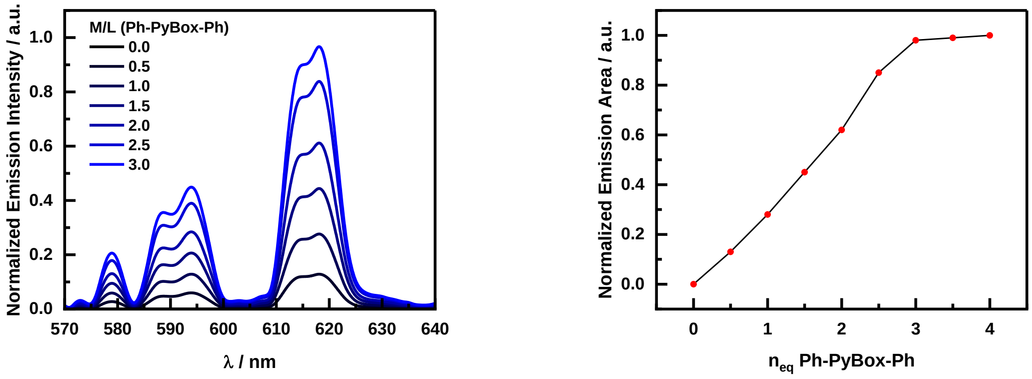

Figure S2. Left: The normalized emission intensity of $Eu(OTf)_3$ with various equivalents of Ph-PyBox-Ph. Right: The isotherm titration curve from the normalized emission integrated area at varying equivalents of Ph-PyBox-Ph.

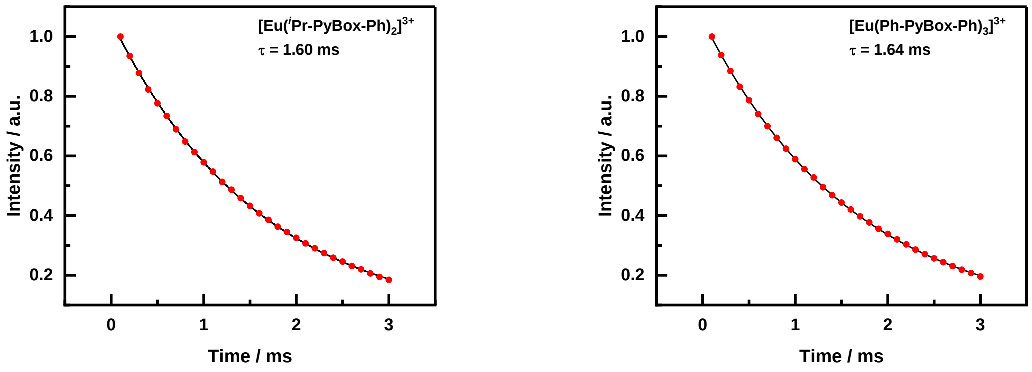

Figure S3. Decay curves of the Eu(III) emissive state along mono-exponential fitting for $[Eu(^{i}Pr\text{-}PyBox\text{-}Ph)_2]^{3+}$ (left) and $[Eu(Ph\text{-}PyBox\text{-}Ph)_3]^{3+}$ (right). Measurements were carried out in 0.01 mM ACN solutions at room temperature.

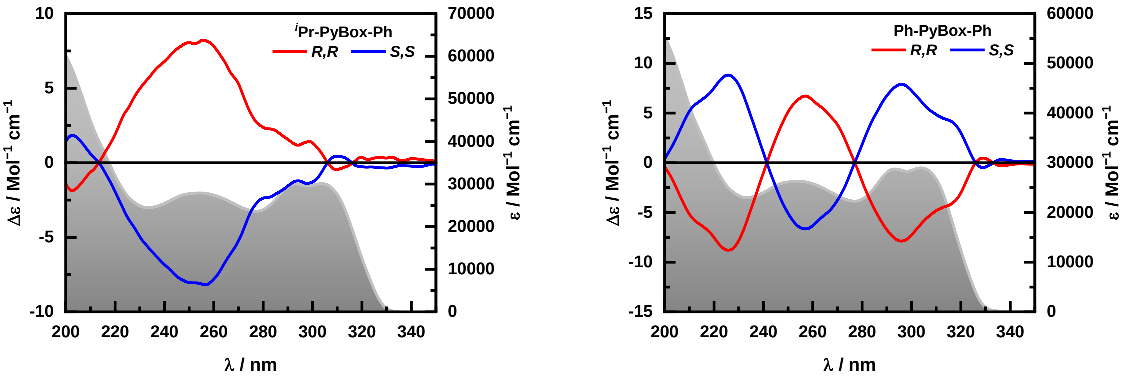

Figure S4. The ECD spectra for each enantiomer and absorption spectrum traced in the background of the ligands ^{i}Pr-PyBox-Ph (left) and Ph-PyBox-Ph (right). Spectra were recorded in 1 mM acetonitrile solutions at room temperature with 0.01 cm cell length. Red solid line: *R*,*R* enantiomer, blue solid line: *S*,*S* enantiomer.

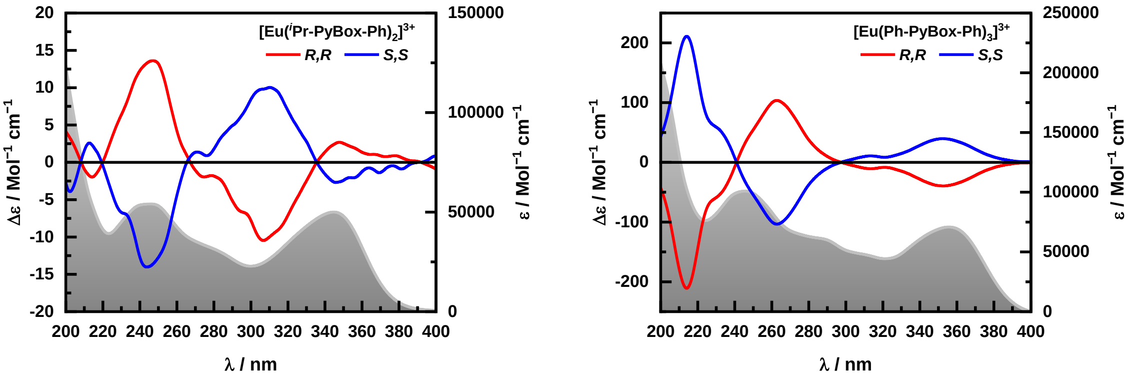

Figure S5. The ECD spectra for each enantiomer and absorption spectrum traced in the background of the complexes [Eu(^{i}Pr-PyBox-Ph)$_2$]$^{3+}$ (left) and [Eu(Ph-PyBox-Ph)$_3$]$^{3+}$ (right). Spectra were recorded in 1 mM acetonitrile solutions at room temperature with 0.01 cm cell length. Red solid line: *R*,*R* enantiomer, blue solid line: *S*,*S* enantiomer.

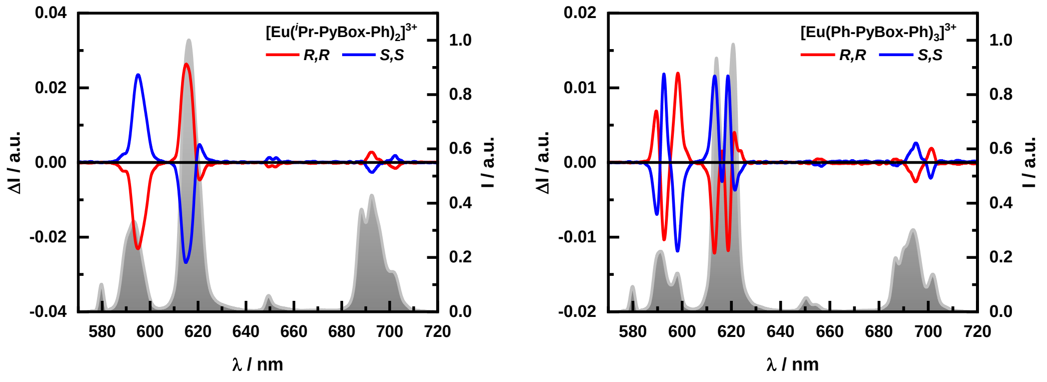

Figure S6. 1PE-CPL spectra of [Eu(^{i}Pr-PyBox-Ph)$_2$]$^{3+}$ (left) and [Eu(Ph-PyBox-Ph)$_3$]$^{3+}$ (right) with the normalized total emission traced in the background for both. Spectra were recorded in 0.01 mM acetonitrile solutions at room temperature under 340 nm excitation. Red solid line: *R,R* enantiomer, blue solid line: *S,S* enantiomer.

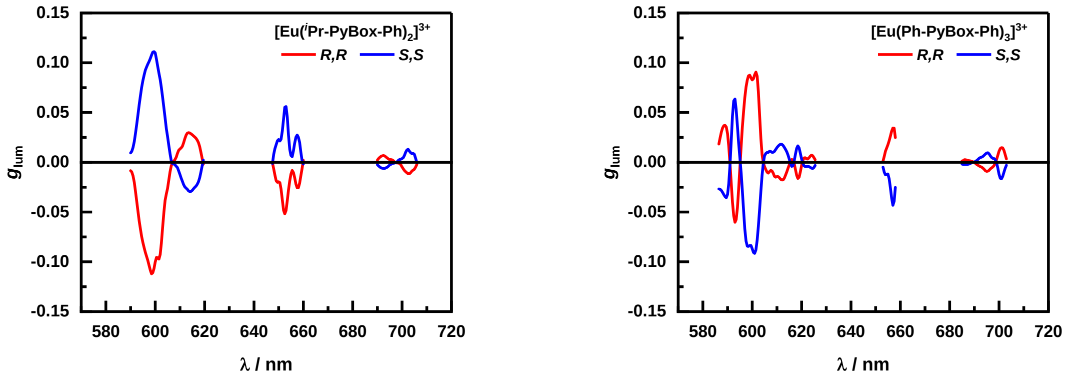

Figure S7. Plots of g_{lum}-vs-wavelength for the 1PE-CPL spectra of [Eu(^{i}Pr-PyBox-Ph)$_2$]$^{3+}$ (left) and [Eu(Ph-PyBox-Ph)$_3$]$^{3+}$ (right).

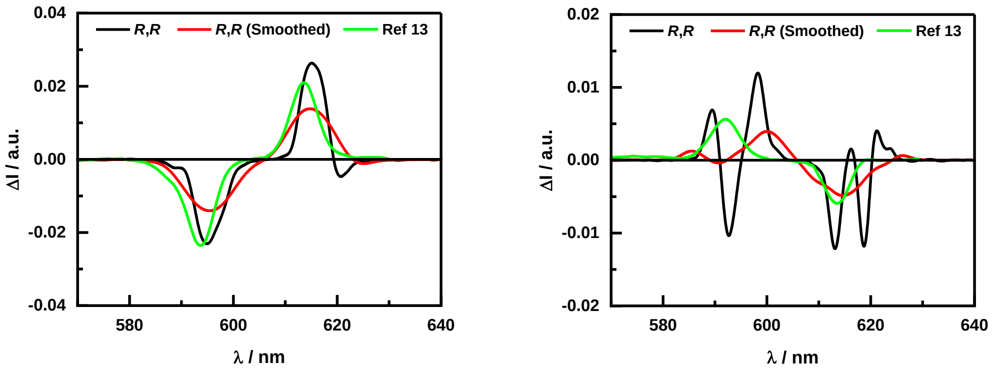

Figure S8. A comparison of the 1P-CPL spectra between the complexes [Eu((*R*,*R*)-^{i}Pr-PyBox-Ph)$_2$]$^{3+}$ (left, black line) and [Eu((*R*,*R*-Ph-PyBox-Ph)$_2$]$^{3+}$ (right, black line), with [Eu((*R*,*R*)-^{i}Pr-PyBox)$_2$]$^{3+}$ and [Eu((*R*,*R*-Ph-PyBox)$_2$]$^{3+}$ (green line), reproduced from Ref [13]. The red lines represent the black line smoothed using an adjacent-averaging method over 26 points, to account for the difference spectral resolution achieved in the present work and in Ref [13].

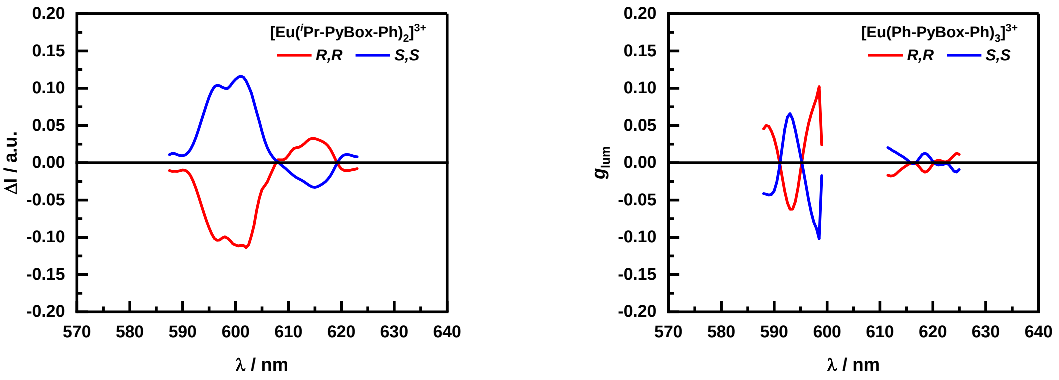

Figure S9. Plots of g_{lum}-vs-wavelength for the 2PE-CPL spectra of $[Eu(^{i}Pr\text{-}PyBox\text{-}Ph)_2]^{3+}$ (left) and $[Eu(Ph\text{-}PyBox\text{-}Ph)_3]^{3+}$ (right).

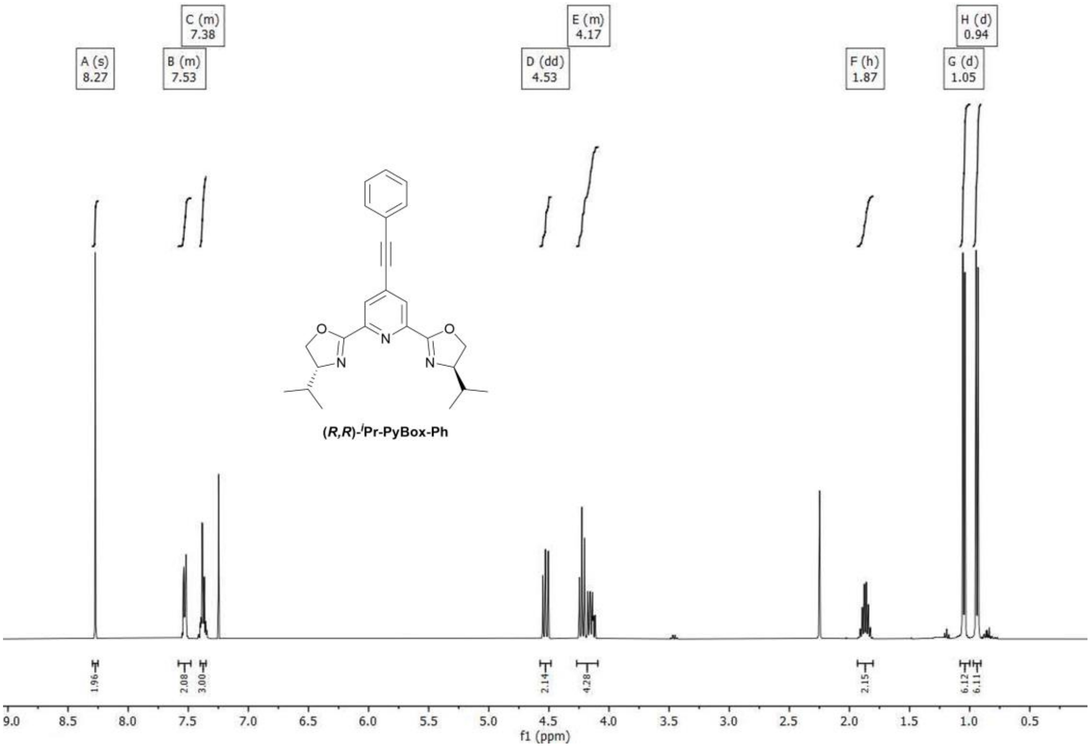

Figure S9. ^{1}H NMR (400 MHz, $CDCl_3$) spectrum of (*R*,*R*)-^{i}Pr-PyBox-Ph.

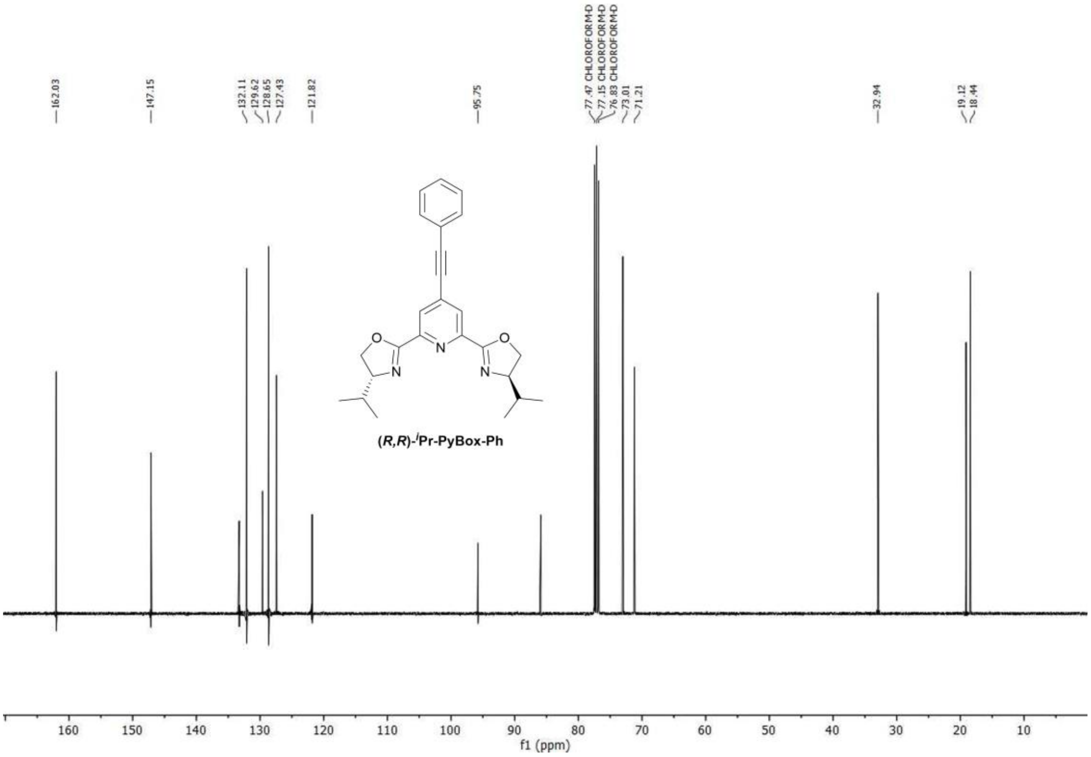

Figure S10. ^{13}C NMR (101 MHz, $CDCl_3$) spectrum of (*R*,*R*)-^{i}Pr-PyBox-Ph.

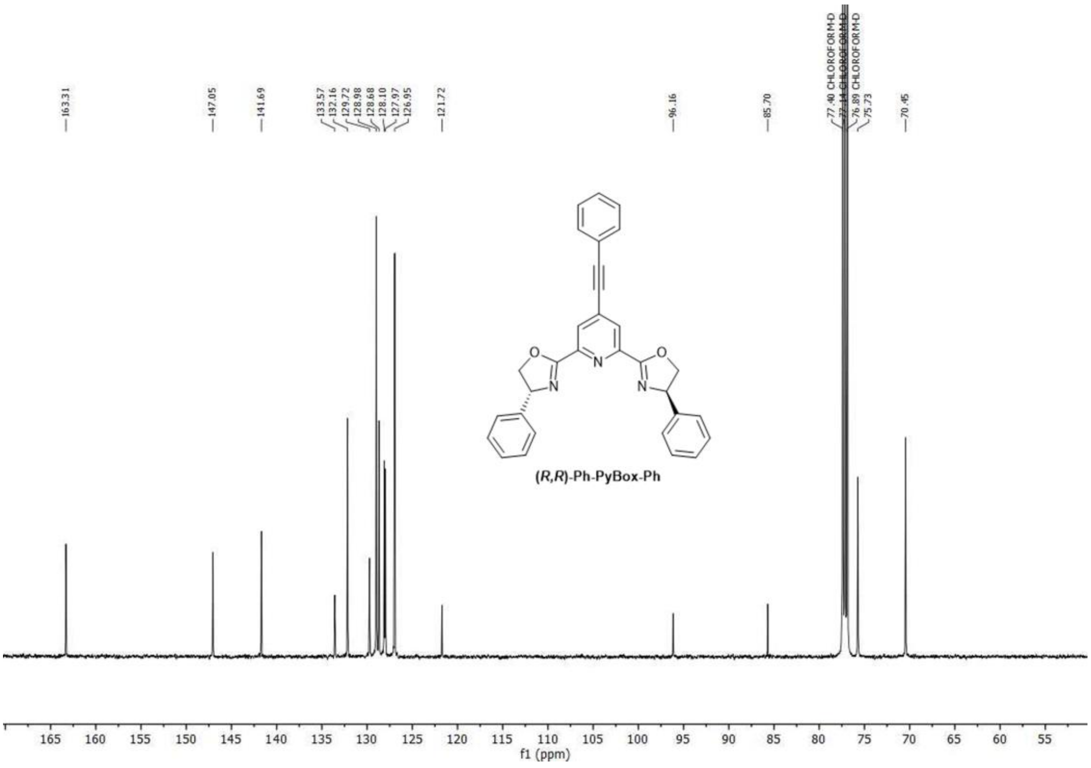

Figure S11. ^{1}H NMR (400 MHz, $CDCl_3$) spectrum of (*R*,*R*)-Ph-PyBox-Ph.

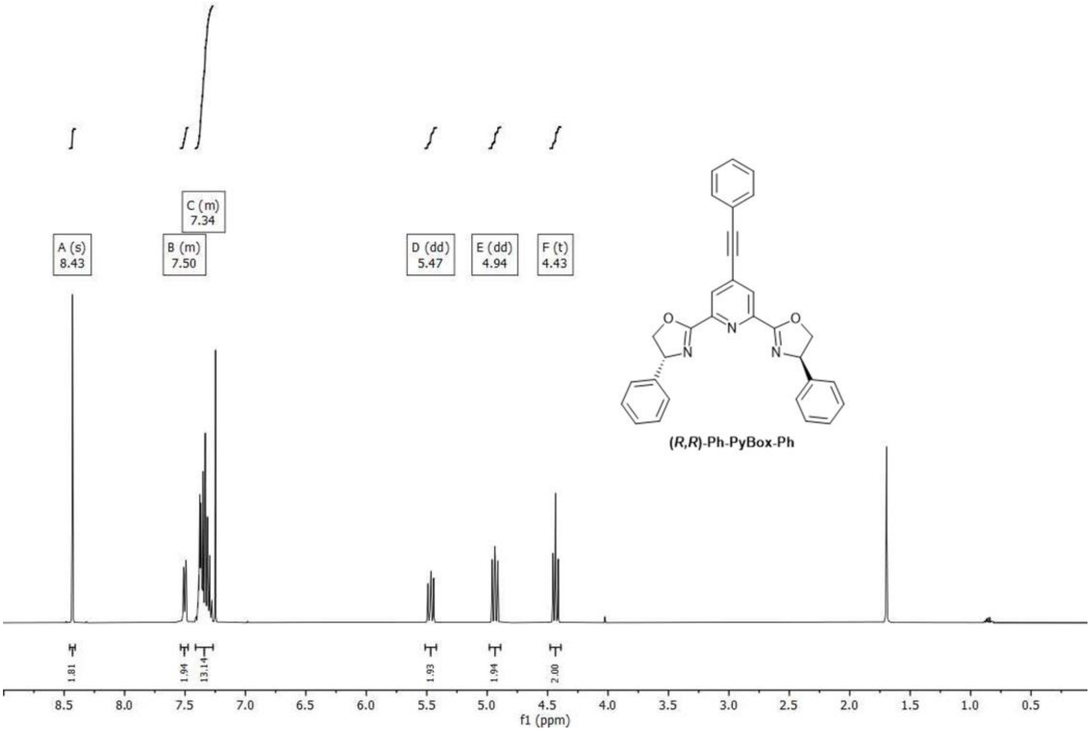

Figure S12. ^{13}C NMR (126 MHz, $CDCl_3$) spectrum of (*R*,*R*)-Ph-PyBox-Ph.

References

1 H. E. Gottlieb, V. Kotlyar and A. Nudelman, *J. Org. Chem.*, 1997, **62**, 7512–7515.

2 J. H. S. K. Monteiro, A. De Bettencourt-Dias and F. A. Sigoli, *Inorg. Chem.*, 2017, **56**, 709–712.

3 O. G. Willis, F. Petri, G. Pescitelli, A. Pucci, E. Cavalli, A. Mandoli, F. Zinna and L. Di Bari, *Angew. Chem. Int. Ed.*, 2022, **61**, e202208326–e202208326.

4 A. Cornejo, J. M. Fraile, J. I. García, E. García-Verdugo, M. J. Gil, G. Legarreta, S. V. Luis, V. Martínez-Merino and J. A. Mayoral, *Org. Lett.*, 2002, **4**, 3927–3930.

5 S. Lundgren, S. Lutsenko, C. Jönsson and C. Moberg, *Org. Lett.*, 2003, **5**, 3663–3665.

6 O. Storm and U. Lüning, *Eur. J. Org. Chem.*, 2003, **2003**, 3109–3116.

7 K. Rurack and M. Spieles, *Anal. Chem.*, 2011, **83**, 1232–1242.

8 R. Carr, R. Puckrin, B. K. McMahon, R. Pal, D. Parker and L.-O. Pålsson, *Methods Appl. Fluoresc.*, 2014, **2**, 024007.

9 P. Stachelek, L. MacKenzie, D. Parker and R. Pal, *Nat. Commun.*, 2022, **13**, 553–553.

10 C. Xu and W. W. Webb, *J. Opt. Soc. Am. B*, 1996, **13**, 481.

11 L.-O. Pålsson, R. Pal, B. S. Murray, D. Parker and A. Beeby, *Dalton Trans.*, 2007, 5726.

12 N. S. Makarov, M. Drobizhev and A. Rebane, *Opt. Express*, 2008, **16**, 4029.

13 L. Arrico, C. Benetti and L. Di Bari, *ChemPhotoChem*, 2021, **5**, 815–821.

Author contributions

O.G.W. carried out spectroscopic measurements, data analysis and assisted in the synthesis of both ligands and complexes; F.P. carried out the synthesis of both ligands and complexes under the supervision of A.M.; F.Z., L.D.B. and RP conceived the idea, supervised the whole work, and discussed the data regularly; RP built and provided the set-up for 2PE experiments and D.F.D.R and R.P assisted with all 2PE experiments. O.G.W. and F.Z. wrote the manuscript. All authors read and approved the final manuscript.

Conflict of Interest

The authors declare no conflict of interest.
